# A small XY chromosomal region explains sex determination in wild dioecious *V. vinifera* and the reversal to hermaphroditism in domesticated grapevines

**DOI:** 10.1186/s12870-014-0229-z

**Published:** 2014-09-03

**Authors:** Sandrine Picq, Sylvain Santoni, Thierry Lacombe, Muriel Latreille, Audrey Weber, Morgane Ardisson, Sarah Ivorra, David Maghradze, Rosa Arroyo-Garcia, Philippe Chatelet, Patrice This, Jean-Frédéric Terral, Roberto Bacilieri

**Affiliations:** Centre de Bio-Archéologie et d’Ecologie CBAE (UMR 5059 CNRS/Université Montpellier 2/EPHE/INRAP). Equipe Interactions, Biodiversité, Sociétés, Institut de Botanique, 163 rue Auguste Broussonet, 34090 Montpellier, France; INRA, UMR 1334 AGAP, Equipe Diversité, Adaptation et Amélioration de la Vigne, F34060 Montpellier, France; Institute of Horticulture, Viticulture and Oenology, Agrarian University of Georgia, University Campus at Digomi, David Aghmashenebeli Alley, 13-th km. 0159, Tbilisi, Georgia; CBGP-INIA. Dpto Biotecnología, Campus de Montegancedo, Autovía M40, km38, 28223 Pozuelo de Alarcón, Madrid Spain; Université Montpellier 2, Place Eugène Bataillon, 34095 Montpellier, France

**Keywords:** Dioecy, Domestication, Hermaphroditism, Sex chromosome, *Vitis vinifera* L

## Abstract

**Background:**

In *Vitis vinifera* L., domestication induced a dramatic change in flower morphology: the wild *sylvestris* subspecies is dioecious while hermaphroditism is largely predominant in the domesticated subsp. *V. v. vinifera*. The characterisation of polymorphisms in genes underlying the sex-determining chromosomal region may help clarify the history of domestication in grapevine and the evolution of sex chromosomes in plants. In the genus *Vitis,* sex determination is putatively controlled by one major locus with three alleles, male *M*, hermaphrodite *H* and female *F,* with an allelic dominance *M* > *H* > *F.* Previous genetic studies located the sex locus on chromosome 2. We used DNA polymorphisms of geographically diverse *V. vinifera* genotypes to confirm the position of this locus, to characterise the genetic diversity and traces of selection in candidate genes, and to explore the origin of hermaphroditism.

**Results:**

In *V. v. sylvestris*, a sex-determining region of 154.8 kb, also present in other *Vitis* species, spans less than 1% of chromosome 2. It displays haplotype diversity, linkage disequilibrium and differentiation that typically correspond to a small XY sex-determining region with XY males and XX females. In male alleles, traces of purifying selection were found for a *trehalose phosphatase*, an *exostosin* and a *WRKY transcription factor*, with strikingly low polymorphism levels between distant geographic regions. Both diversity and network analysis revealed that *H* alleles are more closely related to *M* than to *F* alleles.

**Conclusions:**

Hermaphrodite alleles appear to derive from male alleles of wild grapevines, with successive recombination events allowing import of diversity from the X into the Y chromosomal region and slowing down the expansion of the region into a full heteromorphic chromosome. Our data are consistent with multiple domestication events and show traces of introgression from other Asian *Vitis* species into the cultivated grapevine gene pool.

**Electronic supplementary material:**

The online version of this article (doi:10.1186/s12870-014-0229-z) contains supplementary material, which is available to authorized users.

## Background

The wild grapevine, *Vitis vinifera* L. subsp. *sylvestris*, is the wild ancestor of the domesticated grapevine *V. v. vinifera* [1,2], cultivated for wine and table grape production [3]. The genus *Vitis*, a monophyletic taxon of the family *Vitaceae* [4,5], includes approximately sixty species present mainly in Asia and America, all of which -except the domesticated grapevine- are dioecious (male and female flowers borne on different plants) [6,7]. During grapevine domestication, flower reproductive morphology has incurred radical modifications, with the change from dioecy to hermaphroditism in domesticated grapevines [8]. The geographic origin of hermaphroditism development in the domesticated grapevine is still not elucidated, nor is it known whether it occurred during primary [1,9] and/or secondary domestication events believed to have occurred in geographically distinct areas around the Mediterranean [10,11].

Sex expression in *Vitis* flower is thought to be controlled by a major locus with three alleles, male *M*, hermaphrodite *H* and female *F,* with an *M* > *H* > *F* allelic dominance [6,7,12–14]. Several genetic maps based on interspecific crosses have confirmed that sex determinism in the genus *Vitis* is under the control of a single major genomic region located on chromosome 2, close to the SSR marker VVIB23 [15–17]. Recently, a complex interspecific cross (*V. vinifera* x [*V. riparia* x *V. cinerea*]) was used by Fechter et al. [18] to narrow the location of the sex locus to a 143 kb genomic region located between positions 4.907.434 and 5.037.597 bp of chromosome 2 [18] on the physical map of the *V. vinifera* reference genome sequence (PN40024 12x.0 version [19]). So far, the co-localisation on chromosome 2 of the sex locus in *V. vinifera* subsp. *vinifera* has been confirmed only in the genetic map of one intra-specific cross [20], with a recombination distance of 0.4 cM from the nearest genetic marker (VVIB23). Moreover, in the *V. v. sylvestris* subspecies, the sex locus localisation remains to be confirmed.

The evolution of proper sex chromosomes is quite rare in plants: indeed, approx. 40 species of flowering plants are currently known to have developed sex chromosomes and among them, half have heteromorphic sex chromosomes [21]. A sex chromosome may start to develop in dioecious species through the suppression of recombination between male- and female-sterile mutations with complementary dominance in close proximity on a chromosome [22]. Then, this sex-determination region would gradually grow in size, increasingly incorporating sex-linked genes and eventually evolving into heteromorphic sex chromosomes [21,22]. Some of the processes involved in sex chromosome evolution, as the suppression of genetic recombination or the genetic degeneration of the Y chromosome, are not well understood and only the study of the sex-determining systems on different species and at different steps of evolution could provide some answers [23]. While the sex determination locus in *Vitis* species was mainly studied to develop genetic markers for early sexing for breeding purposes [18,20], the work of Fechter et al. [18], evidencing a small sex-determination region, suggests that *Vitis* species could be good candidates to study the early steps of sex chromosome evolution.

In the present study, we explore the sequence polymorphisms near the sex locus in a genetically and geographically diverse panel of wild and domesticated grapevines, with the objectives to: i) confirm the position and boundaries of the sex locus in *V. vinifera* subsp. *sylvestris*; ii) characterise the sex region in terms of linkage disequilibrium, genetic diversity, selection signature and candidate genes; and iii) use this information to explore the geographic and genetic origin of hermaphroditism in domesticated grapevine.

Since wild grapevines carry the ancestral form of the sex locus from which the domesticated grapevine hermaphroditism derived, we first mapped sequence polymorphisms linked to the sex trait in *Vitis vinifera* subsp. *sylvestris*. Then, we compared the polymorphisms linked to the sex trait in diverse wild and domesticated grapevine populations to study the origin of hermaphroditism in domesticated grapevines.

## Methods

### Plant material and phenotypic trait data

The plant material consisted of 73 wild (39 females and 34 males) and 39 hermaphrodite domesticated grapevines (Additional file 1). These grapevines were chosen among 139 wild genotypes and 2.323 domesticated genotypes [24] to maximise both genetic diversity and geographic representation. Three genotypes from other species were also considered to represent genetic variation in the subgenus *Vitis*: *V. balansaeana*, *V. coignetiae* and *V. monticola* [25]. The grapevines were sampled either in natural populations or from the French National Grapevine Germplasm Collection (INRA, Domaine de Vassal, France; http://www1.montpellier.inra.fr/vassal/). The genotypes considered varied according to the genetic analyses (Additional file 1). Sex phenotypes (male, female or hermaphrodite) were evaluated by observations of flower morphology repeated over several years, and coded according to the International Organization of Vine and Wine descriptors (code number OIV-151 [26]).

### DNA extraction

DNA was extracted from 150 mg of leaves according to the Dneasy Plant Mini Kit (Qiagen) instructions with 1% Polyvinylpyrrolidone (PVP 40.000) and 1% of β-mercaptoethanol added to the buffer AP1 to eliminate polyphenols, strong inhibitors of in-vitro enzymatic reactions abundantly present in the crude grape cell lysate.

### Amplicons sequencing

**Figure 1:**
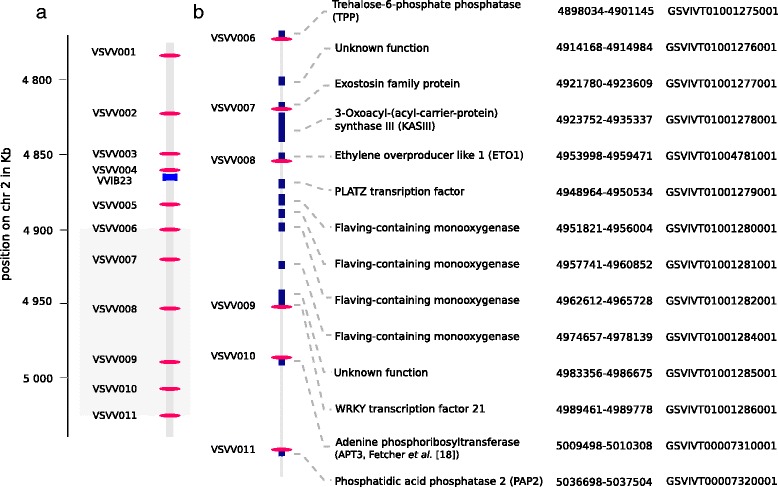
**Amplicon position in the sex locus and its boundaries on chromosome 2 of the 12×.0 reference sequence version. a)** VVIB23 SSR marker (light blue rectangle) and amplicon position (red ellipses); **b)** Amplicon position and gene Gaze annotation in the 143 kb sex locus defined by Fechter et al. [18]. The 12×.0 annotated genes version are represented in dark blue and our amplicons in red. For the APT3 and the ETO1gene, we used the synteny between the chromosome 2 of the 8X reference sequence version, the unassembled scaffold_233 of the 12×.0 reference sequence version, and the BAC sequencing maps of *V. riparia* and *V. cinerea* [18] to estimate their relative position on chromosome 2, 12×.0 version (see Methods). The phosphatidic phosphatase 2 (PAP2), is not predicted by the Gaze annotation of the 12×.0 reference sequence version but it is annotated by the Gaze annotation of the 8× reference sequence version and confirmed by Fechter et al. [18] on the 12×.0 version.

Several studies located the sex locus in *Vitis* close to the SSR marker VVIB23 on chromosome 2 [15–17,20,27]. In addition, we preliminary confirmed this locus in *Vitis vinifera* subsp. *sylvestris*, using 11 SSR markers segregating in several intra-specific crosses resulting from open-pollination (data not shown). Using this information, we designed 11 amplicons to cover a region between positions 4.781.551 bp and 5.037.597 bp of chromosome 2 (PN40024 grapevine genome reference sequence, version 12×.0 [19]; Table 1, Additional file 2 for primer sequences). This region covers both the VVIB23 SSR marker and the 143 kb region as defined by Fechter et al. [18] (Figure 1). We did not extend the coverage further downstream as we found that the SSR marker VMC3b10 (position 5.057.413 bp) was not associated with sex segregation in our wild grapevine mapping populations (data not shown).

**Table 1:** **Characteristics of the amplicons used in this study to cover the sex locus and its edges**

**Amplicon name**	**Position**	**Amplicon size**		Gene annotation*
			Name	Putative function
VSVV001	4781551 - 4782603	1053	GSVIVT01004916001	Esterase/lipase/thioesterase family protein
VSVV002	4822617 - 4824068	1452	GSVIVT01001263001	SAUR family protein
VSVV003	4850582 - 4851997	1416	GSVIVT01001267001	Pentapeptide repeat protein
VSVV004	4861475 - 4862891	1417	GSVIVT01001269001	Yabby14 protein
VSVV005	4883461 - 4884818	1358	GSVIVT01001272001	Soluble acid invertase
VSVV006	4900275 - 4901493	1219	GSVIVT01001275001	Trehalose-6-phosphate phosphatase (TPP)
VSVV007	4921838 - 4923352	1515	GSVIVT01001277001	Exostosin family protein
VSVV008^†^	4953195 – 4954179**	984	GSVIVT01004781001	Ethylene Overproducer-like 1 (ETO1)
VSVV009	4989467 – 4990268	802	GSVIVT01001286001	WRKY transcription factor 21
VSVV010^‡^	5009549– 5010222**	673	GSVIVT00007310001	Adenine phosphoribosyltransferase (APT3)
VSVV011^§^	5036645 – 5037597	953	GSVIVT00007312001	Phosphatidic acid phosphatase 2 (PAP2)

*Gaze annotation, **Approximative values. ^†^PN40024 reference sequence, 12×.0 version, amplicon position 16.072.323-16.073.307, Scaffold_233, chromosome UnRandom; ^‡^PN40024 reference sequence, 8× version, amplicon position 5.192.572-5.193.382, scaffold 187, chromosome 2; ^§^Primers developed in the gene predicted using the 8× Gaze annotation and confirmed by Fechter et al. [18] on the 12×.0 reference sequence version.

According to Fechter et al. [18], the 143 kb region of chromosome 2 (12×.0 version) between 4,907,434 and 5,050,616 bp corresponds to the female allele of the hermaphroditic Pinot Noir 40024, while the slightly different hermaphrodite allele is located on the unassigned scaffold_233 (chromosome UnRandom of the 12×.0). The 12×.0 scaffold_233 is collinear with the chromosome 2 of the 8× grape genome reference sequence [19]; both these assemblies display two regions which are absent from the chromosome 2 assembly of the 12×.0 reference sequence version: a region between the *3-Oxoacyl synthase III C terminal* (KASIII) and the *PLATZ transcriptor factor*, and the *adenine phosphoribosyl transferase* (APT3) region [18]. The APT3 distinguishes female individuals from male and hermaphroditic ones [18]. A gene, the *phosphatidic phosphatase 2* (PAP2), is not predicted by the Gaze annotation of the 12×.0 reference sequence version but it is annotated by the Gaze annotation of the 8× reference sequence version and confirmed by Fechter et al. [18] on the 12×.0 reference sequence version.

For our work, eight primer pairs out of the eleven could thus be designed using the Gaze annotation of the 12×.0 reference sequence version (Table 1, Figure 1). A primer pair (VSVV011) was developed in the PAP2 gene using the Gaze annotation of the 8× reference sequence version (Table 1). Another primer pair (VSVV010) was specifically developed to cover the region of the putative APT3 distinguishing female individuals from male and hermaphroditic ones [18]. A last amplicon (VSVV008) was designed to amplify a gene present in the region between the KASSIII and the PLATZ transcriptor on the 12×.0 scaffold_233; the predicted protein of this gene blasts with an *Ethylene Overproducer-like 1* gene (ETO1, blastx E-value = 4e-83). For the ETO1 and APT3 amplicons, the positions on the grape genome physical maps were estimated based upon a manual realignment of the unassigned scaffold_233 (chromosome UnRandom of the 12×.0) and the 8× reference sequence version respectively, on the chromosome 2 of the 12×.0 reference sequence version. As a consequence, in our work the 12×.0 positions of these two amplicons are approximate (Table 1).

All primer pairs were designed using the Primer3 software V.0.4.0 [28,29] so as to amplify stretches between 600 and 1.300 bp and cover a part of the promoter and the first exons and introns [28,29]. Thermocycling consisted of an initial stringent cycle (94°C for 3 minutes followed by 12 cycles of 94°C for 30 seconds, from 65 to 56°C decreasing by 0.70°C at each cycle for 45 seconds, 72°C for 120 seconds) and additional 25 cycles of 94°C for 30 seconds, 56°C for 45 seconds, 72°C for 90–120 seconds. Sequencing was performed on PCR products purified using the AMPure® kit (Agencourt®, MA, USA); BigDye® Terminator v3.1 Cycle Sequencing Kit (Applied BioSistem, CA, USA) was used following the standard protocol and reaction products were purified with the CleanSEQ® kit (Agencourt) and read on a 3130×l Genetic Analyzer (Applied BioSystems). Raw sequence files (AB1 format) were imported, aligned and trimmed using the Staden software v.2.0.0 [30]; SNP calling was carried out manually using the Staden interface. Then, fasta files were exported and subsequently analysed in other softwares and pipelines.

### Identification of sequence polymorphisms linked to the sex trait

Phenotypic sex inheritance in wild grapevines produces only male and female variants, with a ratio near to 1:1 in adult populations (even if some variation in sex phenotypes have been observed [13,26], in our sample only two morphs were found, M and F). The most parsimonious hypothesis we could make on sex inheritance in grape, based on previous observations, preliminary data analysis, and literature survey [6,7,17,18,20], was that of a XY system, where, at the sex locus, the female is homozygous (XX) and the male is heterozygous (XY).

To map the sex locus on the genome, we first used a genetic association approach, looking for correlations between sex flower phenotypes and sequence polymorphisms in a panel of diverse wild genotypes from different geographic provenances (Additional file 1). However, the use of general or mixed linear models searching for association resulted in too many false positives (SNP that were correlated to sex but explained only a portion of the phenotypes). Thus, we used an approach similar to Siegismund [31], using Fisher tests to compare, for each polymorphism and for male and female wild grapevines separately, the expected and observed proportions of homozygous and heterozygous genotypes. The expected proportions were assumed to follow the Hardy–Weinberg law and were calculated from the allele frequencies observed in the entire population (sum of male and female individuals). The observed counts were the number of homozygous and heterozygous genotypes actually recorded in male and female grapevines. Indels were coded as present/absent (Additional file 3). Fisher tests were calculated with the *fisher.test* function of the R statistical software [32]. We only considered sequence polymorphisms with less than 20% missing data and with a minimum allele frequency in the sample higher than 5%. A test was considered significant when the probability of deviation from the null hypothesis was inferior to a 0.05 P-value threshold adjusted by a Bonferroni correction for multiple hypotheses testing (0.05/n with n corresponding to the total number of studied polymorphisms).

### Linkage disequilibrium in the sex region

To explore linkage disequilibrium between and within amplicons covering the sex region, we used the *Measure.R2VS*() function in the R package LDcorSV [33]. *r*^*2*^*VS* is the square of each pairwise correlation corrected by both the relatedness and genetic structure of the sample [33]. The sample considered here was composed of 18 male and 18 female individuals (Additional file 1). These 36 specimens were chosen among those with the least missing data, eliminating the most closely related individuals and equilibrating their geographic representation. The genetic structure matrix was calculated from a dataset of 20 SSRs [24] of all the wild genotypes in this study, using STRUCTURE software [34]. We used the model with uncorrelated allele frequencies, admixture, and no prior population information, previously showed to be pertinent in grapevine [35]. Ten STRUCTURE runs (each with 5 × 10^5^ iterations and 5 × 10^5^ replicates) for each K-level were obtained and compared to estimate group assignation stability. The most probable number of sub-populations was inferred based on both the similarity pattern among the 10 STRUCTURE replicates and Evanno’s Δks statistics [36]. The kinship matrix was obtained using ML-Relate software [37] with the same SSR markers and genotypes as above.

### Diversity in *M*, *F* and *H* haplotypes and signature of selection

To compare the diversity of male, female and hermaphrodite alleles at the significant sex-linked amplicons (see Additional file 1 for the genotypes considered), the haplotypes were reconstructed using PHASE v2.1 with default parameter values [38,39]. The attribution of individual haplotypes to the *M*, *F* and *H* groups (called hereafter haplogroups) were carried out with the help of haplotype trees (Additional file 4) built with a maximum likelihood method (PhyML 3.0 [40]) implemented in SeaView v4.3.3 [41] and based on the Generalised Time-Reversible (GTR) model [42].

Genetic diversity in *M*, *F* and *H* haplotypes was evaluated with the following statistics: number of haplotypes (Nh), number of segregating sites (S), haplotype diversity (H) and nucleotide diversity (π). In order to detect a signature of selection in the sex region, Tajima’s D [43] and Fu and Li’s D* [44] statistics were calculated with the DnaSP v5 software [45] separately for the male, female and hermaphrodite haplogroups. To confirm traces of selection detected on the male haplogroups with the Tajima’s D and the Fu and Li’s D* tests, the E statistics and the DH test [46] were computed using the male haplotype of *V. balanseana* as an outgroup (Table 2).

**Table 2:** **Allocation of 0, 1 or 2 female haplotypes (** ***F)*** **to the hermaphrodite, male and female genotypes, according to the maximum likelihood trees, for the four sex linked amplicons**

**Genotype**	**VSVV006**	**VSVV007**	**VSVV009**	**VSVV010**
Hermaphrodite	*n = 22*	*n = 22*	*n = 22*	*n = 21*
0 haplotype *F*	1	0	1	9
1 haplotype *F*	21	22	19	8
2 haplotypes *F*	0	0	2	4
Male	*n = 22*	*n = 22*	*n = 22*	*n = 18*
0 haplotype *F*	0	0	0	0
1 haplotype *F*	22	22	22	18
2 haplotypes *F*	0	0	0	0
Female	*n = 24*	*n = 24*	*n = 24*	*n = 22*
0 haplotype *F*	0	0	0	0
1 haplotype *F*	0	0	0	0
2 haplotypes *F*	24	24	24	22

Finally, we evaluated the intraspecific genetic differentiation between male, female and hermaphrodite haplogroups, and the interspecific differentiation between *V. v sylvestris* and *Vitis* species haplotypes, using the Fst statistics [47,48] with DnaSP v5 software as well. The *Vitis* species used for this statistics were *V. balanseana*, *V. monticola* and *V. coignetiae*.

### Origin of the H haplotypes

Combining the haplotypes of the four sex-linked amplicons, the *M*, *F* and *H* macrohaplotypes were reconstructed. PHASE v2.1 was run again using a 100 burn-in period with 100 iterations with a thinning interval of 1 and 10 repeats. The algorithm was run several times, validating convergence. Then, to understand the origin of *H* haplotypes in the domesticated grapevine, a network analysis was carried out on the *F*, *M* and *H* macrohaplotypes using the median-joining method as described in Bandelt et al. [49] and implemented in Network v4.6.1.1. [50]. A Star Contraction was run before running the network calculation.

Finally, the relationship between the network distances (in number of mutations) of the *H* haplotypes from the *M* haplogroup, and the geographic origin, grape use (table, wine or both), degree of domestication (ancient or modern cultivars [51]) and the genetic structure ancestry of the domesticated grapevines [35] were explored using an ANOVA.

## Results

### Sequence polymorphisms linked to the sex trait

**Figure 2:**
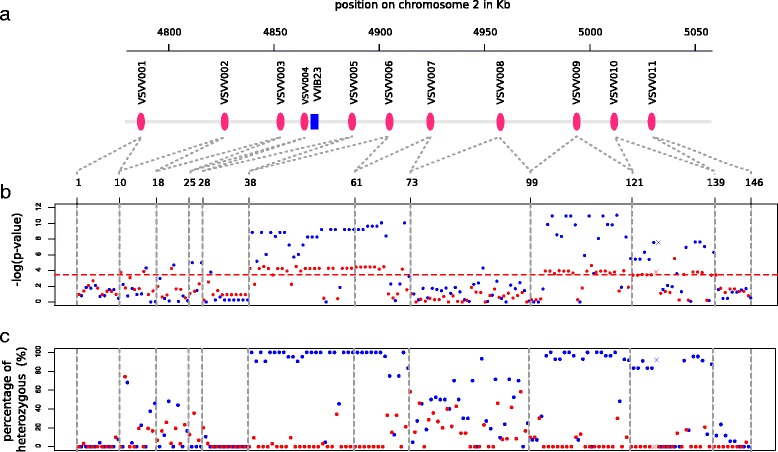
**Polymorphisms in the sex region. a)** Amplicon position along the sex locus on chromosome 2. **b)** Fisher test probabilities of deviation from the expected Hardy-Weinberg genotype proportions in wild grapevines (31 males and 34 females). The significant Fisher tests are represented by dots above the red dashed line, which is the log-transformed Bonferroni threshold (−log(0.05/146) = 3.47). Red dots represent the p-values calculated on female genotypes and blue dots those for males. The vertical dashed lines represent the separations between the amplicon. The coloured crosses in VSVV010 correspond to the sex-linked *indel* found by Fechter et al. [18] in the second intron of the APT3 gene. **c)** Percentage of heterozygous genotypes. The heterozygosity proportions are represented by red dots in the 34 females and by blue dots in the 31 males.

Eleven amplicons representing 9.523 bp in total and designed to partly amplify gene sequences were chosen to cover the sex locus and its boundaries [18,20]. Sequencing these 11 amplicons on a sample of 65 genetically and geographically diverse wild genotypes (31 males and 34 females, Additional file 1, [GenBank: KJ575622-KJ57662]), allowed the identification of 146 polymorphic sites (Additional file 3): 137 SNPs and 9 indels. Thirty-six SNPs were located in introns and twenty in exons, among which ten were non-synonymous. The allele frequencies of 51 and 64 polymorphisms in female and male genotypes respectively were found significantly deviating from the Hardy–Weinberg proportions (Figure 2b). These significant polymorphisms were mainly found in VSVV006, VSVV007, VSVV009 and VSVV010 (87,04% of the significant polymorphisms in females and 90.60% in males).

Among the significant polymorphisms, 28 perfectly fitted the XY sex determination model. For these polymorphisms, 100% of the male genotypes were heterozygous and 100% of the female genotypes were homozygous for the most frequent allele, i.e. for example males were A/T and females were A/A but never T/T (Figure 2c, Additional file 3). In hermaphrodite domesticated genotypes, these same polymorphisms were in the majority of cases in a heterozygous state (Additional file 5). These 28 polymorphisms, perfectly fitting the XY model, were only found in the VSVV006, VSVV007 and VSVV009 amplicons and 3 of them resulted in non-synonymous amino acid changes (38th, 61th and 66th polymorphism in VSVV006 or VSVV007, Additional file 3).

Moreover, 18 significant polymorphisms in VSVV006, VSVV007, VSVV009 and VSVV010 were only slightly deviating from the XY sex determination model, with all female genotypes homozygous for the most frequent allele and one or two non-heterozygous males (Additional file 3). For example, for the polymorphism 126 (crosses in Figure 2b, c) corresponding to the sex-linked indel in the second intron of the APT3 gene [18], all female were homozygous without the indel while 92% of male were heterozygous (23 heterozygous, one homozygous with the indel and one homozygous without it) (Additional file 3). In the VSVV008 amplicon, only one SNP was found slightly deviating from the XY sex determination model (Figure 2b and Additional file 3).

By contrast, and although few of them were found significantly departing from Hardy-Weinberg proportions (Fisher test), the polymorphisms found in VSVV002, VSVV003, VSVV004 and VSVV005, largely deviated from the XY model, particularly in male genotypes (Figure 2 and Additional file 3).

In summary, 46 significant polymorphisms in the VSVV006, VSVV007, VSVV009 and VSVV010 amplicons fitted either strictly (28) or closely (18) the XY sex-determination model. These results allowed us to define the boundaries of the sex locus at the positions 4.884.818 and 5.036.645 on chromosome 2 of the PN40024 physical map (12×.0 version). This 151,83 kb region, externally delimited by the gene fragments VSVV005 and VSVV011 contains 13 candidate genes (Figure 1 and Additional file 6).

### Linkage disequilibrium in the sex region

The intra- and inter-amplicon linkage disequilibrium (LD) was estimated on a sub-sample of 18 male and 18 female wild grapevines (Additional file 1), by calculating the pairwise square correlation coefficient *r*^*2*^*VS* [33], correcting for the structure and kinship of the sample. Only sequence polymorphisms with less than 20% missing data and with a 0.2 minor allele frequency were analysed. At these thresholds, no polymorphisms were retained in the VSVV001 fragment.

**Figure 3:**
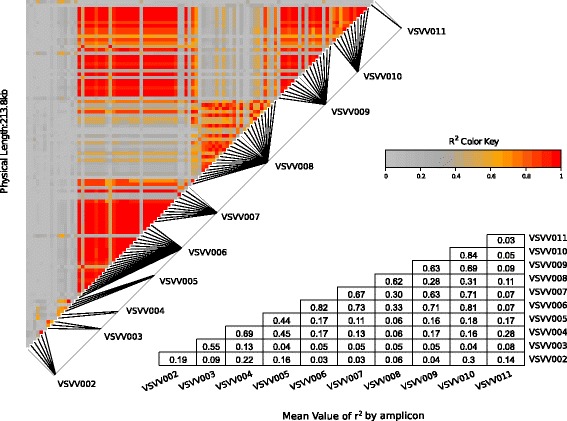
**Linkage disequilibrium plot based on** ***r*** ^***2***^_***VS***_ **values for the SNPs and indels of the sequenced amplicons.** Only polymorphisms with a major allele frequency > 0.2 were used (none were retained in VSVV001 because of this filter). Indels were coded as present/absent. Bottom table: average LD estimates within amplicon and between amplicon pairs.

The highest values of LD were found within and between the four sex-linked fragments (Figure 3). The mean LD for all pairwise comparisons for the four sex-linked fragment was *r*^*2*^*VS* = 0.72 for a total physical length of 109.76 kb. The maximum mean intra-amplicon LD was *r*^*2*^*VS* = 0.84 over 374 bp for VSVV010 and the minimum was *r*^*2*^*VS* = 0.63 over 504 bp for VSVV009. The maximum inter-amplicon LD was *r*^*2*^*VS* = 0.81 between VSVV006 and VSVV010 (109.39 kb) and the minimum was *r*^*2*^*VS* = 0.63 in between VSVV007 and VSVV009 (67.84 kb). The fragment VSVV008 (only weakly linked to sex) presented a significant but lower LD with the sex-linked fragment (*r*^*2*^*VS* = 0.31).

### Diversity of the *M*, *F* and *H* haplotypes and signature of selection

The *M*, *F* and *H* haplotypes for the four sex-associated amplicons (VSVV006, VSVV007, VSVV009 and VSVV010) were assigned using maximum likelihood haplotype trees. According to the XY model and the rules of dominance described for *Vitis* (*M* > *H* > *F* [6,7,12–14]), the haplogroup containing haplotypes from female, male and hermaphrodite genotypes was designated as the female *F* haplogroup (Additional file 4); it is supposed to contain the *F* haplotypes of *FF* females, *MF* males and *HF* hermaphrodites genotypes. By difference, the alternate haplotypes found in male and hermaphrodite genotypes but not present in the *F* haplogroup, were considered as the *M* and the *H* haplotypes respectively (Additional file 4).

For the wild female and male genotypes, the number of *F* haplotypes found in the female haplotype group trees was consistent with the XY sex model (one *F* haplotype in male genotypes and two in females; Table 3). However, some hermaphrodite genotypes presented, for one or two amplicons only (never for the four amplicons simultaneously) either no or two *F* haplotypes. This departure from the sex model was particularly pronounced in VSVV010.

**Table 3:** **Diversity statistics for wild male/female, cultivated hermaphrodite and female haplotypes groups**

	**VSVV006**	**VSVV007**	**VSVV009**	**VSVV010**
	***1111 bp***	***849 bp***	***690 bp***	***498 bp***
**Wild male haplotypes**				
Effective	22	22	22	18
S	5	1	6	12
Nh	3	2	3	6
H	0.18	0.09	0.18	0.72
π	0.00041	0.00011	0.00079	0.00375
Tajima’s D	−1.99 *	−1.16 (ns)	−2.07 *	−1.71 (ns)
Fu and Li’s D*	−2.91*	−1.57 (ns)	−3.23 **	−2.10 +
Zeng et al.’s E	−1.404*	−0.866 (ns)	−0.551(ns)	−0.334 (ns)
DH test (p-value)	0.148 (ns)	0.331 (ns)	0.023 **	0.035**
**Domesticated hermaphrodite haplotypes**				
Effective	22	22	20	26
S	11	2	3	11
Nh	6	3	4	5
H	0.72	0.26	0.36	0.46
π	0.00216	0.00031	0.00091	0.00474
Tajima’s D	−0.71 (ns)	−1.18 (ns)	−0.69 (ns)	−0.60 (ns)
Fu and Li’s D*	0.53 (ns)	−0.63 (ns)	−0.12 (ns)	0 (ns)
**Wild female haplotypes**				
Effective	71	71	71	62
S	13	6	26	19
Nh	12	7	16	17
H	0.69	0.47	0.86	0.72
π	0.00136	0.00170	0.00526	0.00744
Tajima’s D	−1.24 (ns)	0.38 (ns)	−1.01 (ns)	−0.26 (ns)
Fu and Li’s D*	−1.81 (ns)	0.24 (ns)	0.15 (ns)	1.28 (ns)
**Domesticated female haplotypes**				
Effective	21	22	20	16
S	7	4	9	16
Nh	6	3	8	9
H	0.77	0.26	0.77	0.86
π	0.00224	0.00090	0.00500	0.01089
Tajima’s D	0.89 (ns)	−0.85 (ns)	1.24 (ns)	0.49 (ns)
Fu and Li’s D*	0.66 (ns)	1.10 (ns)	0.86 (ns)	0.93 (ns)

S = number of segregating sites, Nh = number of different haplotypes, H = haplotype diversity and π = nucleotide diversity. For the Tajima’s D values, Fu and Li’s D*, Zeng et al.’s E and DH test : “**” indicate a p-value < 0.01, “*” a p-value < 0.05, “+” a p-value < 0.10 and (ns) non-significance. The E statistics and the DH test were computed using the male haplotype of *V. balanseana* as an outgroup.

**Figure 4:**
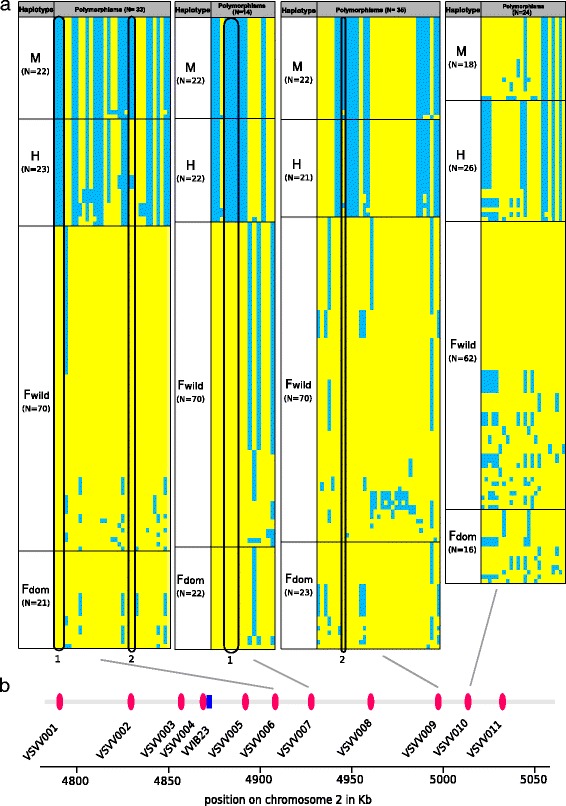
**Sex haplotypes found in the four sex-linked amplicons. a)** Haplotype details by sex : *M* = males, *H* = hermaphrodites, *F wild* = female haplotypes found in wild grapevine, and *F dom* = female haplotypes found in domesticated grapevines. Columns represent the segregating sites in the sex-linked amplicons, with the major allele in yellow and the minor allele in blue. The polymorphisms headed with the number 1 (in black) allow discriminating *F* haplotypes from *H* and *M* haplotypes; those headed with 2 allow differentiating *M* haplotypes from the *H* and *F* haplotypes. **b)** amplicon position on the sex locus on the grapevine chromosome 2.

For diversity parameters calculation and the estimation of selection signature, we differentiated the *F* haplotypes of the hermaphrodite domesticated genotypes from the *F* haplotypes of the male and the female wild genotypes, so as to detect different diversity or selection patterns between the domesticated and the wild compartments. Except for VSVV010, *M* haplogroups presented the lowest number of haplotypes (Nh), and the lowest level of haplotype (H) and nucleotide (π) diversity, revealing the predominant occurrence of one major haplotype, with a low number of SNPs in rare variants (Table 2). The extreme case was the VSVV007 amplicon for which only two haplotypes were observed, differing by only one SNP over 849 bp (polymorphisms n. 3 in Figure 4). On the other hand, in VSVV010, the *M* haplogroups revealed a high haplotype diversity equivalent to the domesticated and wild *F* haplogroups, and a higher π value than for other amplicons (Table 2). The *F* haplogroups of the wild and domesticated genotypes presented strikingly more numerous and diverse haplotypes than the *M* haplogroups. Overall, domesticated and wild *F* haplogroups presented similar diversity patterns.

The *H* haplogroups showed an intermediate diversity pattern between the *M* and *F* haplogroups, but closer to the *M* haplogroups (Table 2). For VSVV010, the *H* haplogroup presented diversity patterns quite equivalent to that of *M* haplogroups, except for a lower haplotype diversity.

To illustrate these findings, the haplotypes identified for each sex-linked amplicon are presented in Figure 4 (for genotype and geographic details see Additional file 7).

This dataset shows that the three grapevine flower sexes, male, female and hermaphrodite, could be correctly predicted in 97% of the genotypes of our geographically representative *V. vinifera* sample, using a few SNPs, i.e. n. 4 to 7 of VSVV007 and n. 8 of VSVV010 (identified by black circles respectively termed 1 and 2 in Figure 4a).

Male haplogroups revealed significantly negative Tajima’s D, and Fu & Li’s D* values for VSVV006 and VSVV009 (Table 2). For VSVV010, the Fu and Li’s D* statistics were close to the significant threshold (0.10 > p-value > 0.05). For male haplogroups (Table 2), all amplicon revealed negative E values, but only VSVV006 showed a significant excess of low-frequency variants. The DH tests detected significantly positive selection on VSVV009 and VSVV010. No other sex haplogroup showed significant signature of selection.

The Fst values (Table 4) revealed a wide genetic distance between the *M* and *F* haplogroups for the four sex-linked amplicons, though less pronounced for VSVV010. The *H* haplogroups were genetically closer to *M* than to *F* haplogroups. For VSVV007, the *H* and *M* haplogroups bore identical haplotypes, thus displaying a null distance. Comparatively, slight genetic differences only were found between the wild and the domesticated *F* haplogroups in VSVV006, VSVV009 and particularly VSVV010. However, for VSVV007, the wild and the domesticated populations of *F* haplogroups seem to be distinct. All genetic differentiation values were lower in VSVV010, revealing that all sex haplogroups are less differentiated in this region. For the four amplicons, the intra-specific genetic distances between male (or hermaphrodites) and female haplogroups were largely superior to the interspecific genetic distance between *Vitis* sp. haplotypes (Table 4).

**Table 4:** **Fst values between combinations of the four sex haplotype groups**

**Haplotype groups**	**Fst**			
	**VSVV006**	**VSVV007**	**VSVV009**	**VSVV010**
*Vitis vinifera* intraspecific comparaison				
Wild males vs. wild females	0.95	0.93	0.88	0.62
Wild males vs. domesticated hermaphrodites	0.62	0.00	0.61	0.54
Domesticated hermaphrodites vs. wild females	0.90	0.92	0.86	0.67
Wild females vs. domesticated females	0.17	0.62	0.16	0.08
*Vitis* sp. vs *Vitis vinifera sylvestris*	0.16	0.04	0.05	0.19

The *Vitis* species used for the interspecific statistics were *V. balanseana*, *V. monticola* and *V. coignetiae*.

### Origin of the H allele

**Figure 5:**
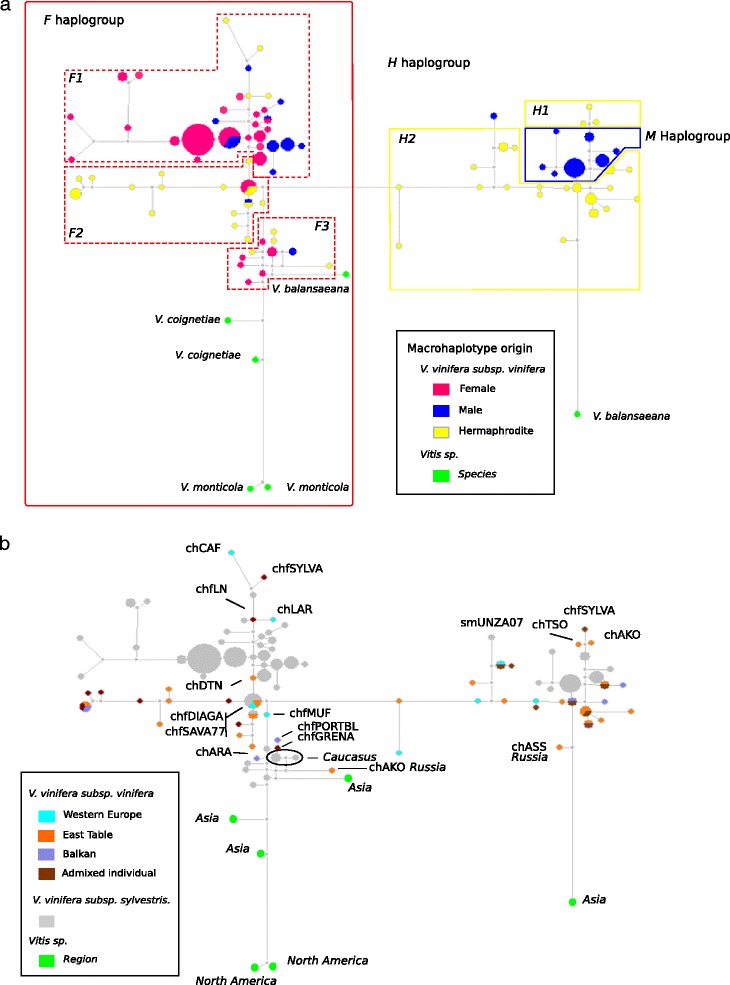
**Consensus network carried out on the** ***F*** **,** ***M*** **and** ***H*** **macrohaplotypes.** Coloured circles regroup together identical haplotypes, with size proportional to their numbers. The distance between pairs of genotypes is proportional to the number of mutations between them. **a)** Pie colours indicate the proportion of phenotypic sex morphs within the group (see legend). Polygons regroup the sex macrohaplotypes; for example, the F haplogroup regroups the 2 female macrohaplotypes of the female genotypes plus the single F macrohaplotype of the males and of the hermaphrodites. **b)** Pie colours indicate the STRUCTURE group of Bacilieri et al. [35]. The shortened name of some hermaphrodite domesticated grapevines are indicated (Additional file 1) as an example.

To determine the origin of the *H* allele, a network was built based on *F*, *M* and *H* macrohaplotypes, combining information provided by the four sex-linked amplicons (Figure 5a). According to this haplotype network, where the distance between pairs of genotypes is proportional to the number of mutations between them, *H* macrohaplotypes were closer to the *M* ones than to the *F* macrohaplotypes. The network displayed two groups of *H* macrohaplotypes: the first (*H1*), at the edge of the network, was only composed of three domesticated grapevines: *cv. Tsolikouri (chTSO), cv. Ak ouzioum Tapapskii (chAKO)* and *cv. Sylvaner (chfSYLVA)*, while the second, *H2* grouped all the others *H* macrohaplotypes of the domesticated hermaphrodite grapevines. The *M* macrohaplotypes of the wild male grapevines were located between the two *H* macrohaplotypes groups. However, one male wild macrohaplotype, *Lambrusque Ul any nad Zitavou A07 (smUNZA07)* from Slovakia, displayed a macrohaplotype closer to the *H2* macrohaplotypes than to the other *M* macrohaplotypes (Figure 5a). This grapevine displayed a VSVV007 haplotype not found in other wild male grapevines, but found in two domesticated hermaphrodite genotypes. Concerning the *F* macrohaplotypes, 3 subgroups could be defined according to the occurrence of wild or domesticated macrohaplotypes (Figure 5a,b). The *F1* group was composed by a majority of wild macrohaplotypes together with 4 cultivars: *cv. Cabernet franc (chCAF)*, *cv. Sylvaner (chfSYLVA)*, *cv. Lignan (chfLN)* and *cv. Lameiro (chLAR)*. The *F2* group contained mostly domesticated macrohaplotypes. In this group, some domesticated grapevines had two identical haplotypes allocated to the *H* haplogroup in the VSVV010; the macrohaplotypes closest to the *F1* and *F3* groups are *cv. Dattier noir (chDTN)*, *cv. Muscat à petits grains blanc (chMUF)*, *cv. Diagalves (chfDIAGA)* and *cv. Savagnin (chfSAVA77)*. A last group *F3* grouped in approximately the same proportions wild and domesticated macrohaplotypes, among which *cv. Portugais bleu (chfPORTBL)*, *cv. Grenache (chfGRENA)*, *cv. Ak ouzioum tpapskii (chAKO)* and *cv. Araklinos (chfARA)*.

To better understand the origin of the *H* alleles, we explored the relationship between the network distances of the *H* macrohaplotypes from the *M* ones (Figure 5b) and the geographic origin, use and degree of domestication [51] of the cultivated grapevines. None of these characteristics revealed a clear correlation with *H* macrohaplotypes positions in the phylogenic network. We then tried to match the network distances with the STRUCTURE groups defined in Bacilieri et al. [35]. This work, based on 2.096 domesticated genotypes, has revealed four main genetic groups: a) wine cultivars from Western regions, b) table grape cultivars from Eastern Mediterranean, Caucasus, Middle and Far East countries, c) wine cultivars from the Balkans and East Europe, and d) a large group of cultivars with admixed genomes. Here, ANOVA analysis revealed a weak tendency (*r*^*2*^ = 0.15, *p* = 0.09) for the Balkan and East Europe cultivars macrohaplotypes, as compared to wine Western cultivars, to be closer to the wild M macrohaplotypes.

Similarly, although pointing to different “degrees of domestication”, the 3 groups of F macrohaplotypes defined above did not show a clear geographic or genetic structure pattern that could explain group composition.

The network position of the macrohaplotypes of the two female *V. monticola*, *V. coignetiae* and the male *V. balansaeana* grapevines used as outgroups, were distributed coherently to their sex phenotype: both macrohaplotypes in the *F* macrohaplogroups for the females, one in the *F* macrohaplogroups and one close to the *M* and *H* macrohaplogroup ones for the male. The closest domesticated macrohaplotypes to the *V. balansaeana* ones belonged to two Russian cultivars : *cv. Assyl kara (chASS)* and *cv. Ak ouzioum tpapskii (chAKO)* (Figure 5).

## Discussion

### Sex region location in *Vitis vinifera* subsp. *sylvestris*

From a locus defined by previous works on inter-specific crosses [17,18], 11 genes were partially sequenced on a diverse set of male and female wild grapevines. Forty-six polymorphisms in four amplicons were found perfectly or strongly linked to flower sex, allowing to locate in *V. v.* subsp. *sylvestris* a sex locus of 151.8 kb on chromosome 2, in full agreement with the 143 kb sex region defined by Fechter et al. [18] on a *Vitis* interspecific cross. Our results corroborates the dominance of the *M* allele over the *F* allele, characteristic of a XY sex-determination model, coherently with sex segregations in controlled crosses [7,12,14]. We also confirmed that the sex locus is situated downstream of SSR marker VVIB23, while previous studies, based on a lower marker density, had placed it upstream [17,20].

Within the 151.8 kb sex region, the polymorphisms of the centrally located VSVV008 amplicon associated only weakly with the sex trait, with one significant SNP only and no perfect M/F association. One hypothesis to explain this pattern may be that local recombination disrupted the association pattern in *V. v. sylvestris*. Unfortunately, in our work we were not able to unequivocally confirm the VSVV008 position within the sex locus. Actually, the PCR primers for this amplicon were designed based on the synteny between several genome sequence assemblies: the chromosome 2 of the 8× grape genome, the putative hermaphrodite allele on the unassigned scaffold_233 (12x.0) and the male *V. cinerea* BAC sequencing map [18], where VSVV008 is located as expected between VSVV007 and VSVV009. According to this information, we expected that VSVV008 would amplify only in males; however, in our *V. sylvestris* sample, it amplified indifferently of sex. Even if the sequence obtained or its PCR primers did not blast anywhere else in the genome some doubts still remain about the true coordinates of VSVV008; only new specifically designed experiments may help to definitely confirm the VSVV008 position.

### Characterisation of the sex locus

Over the four genes linked to sex, we found a strong LD, unprecedented in *Vitis vinifera*, with a mean *r*^*2*^*VS* of 0.72 over 109.76 kb. In *V. vinifera*, LD has been shown to decay rapidly: in more than 200 gene fragments, Lijavetsky et al. [52] observed an LD decay lower than 0.2 at around 200 bp, a finding later confirmed through massive genotyping by Myles et al. [53]. A larger LD in the sex locus, as compared to other genome regions, could be an indication of suppression of recombination, a feature typical of heteromorphic XY-like chromosomal regions [23].

The lowest values of haplotype diversity (H) were found in male haplotypes of wild grape, with the predominant occurrence of one major haplotype, distributed without variation over largely diverse geographic origins, from Eastern to Western European, Caucasian and North African provenances. Hermaphrodite domesticated grapes displayed haplotype diversity values higher than that in wild males, while female haplotypes had the highest values, without notable differences between wild and domesticated pools. The large Fst values between males and females confirm the clear genetic differentiation between the *M* and the *F* haplotypes. The negative significant Tajima’s D and the Fu and Li’s D* values in *M* haplotypes of VSVV006 and VSVV009 indicate an excess of rare polymorphisms, revealing purifying selection. Using *V. balanseana* as outgroup, the Zeng et al. E statistics and DH test [52] confirmed this pattern for VSVV009. For the VSVV007 *M* haplotypes, these statistics were negative but not significant, probably because over its 849 bp length, we found only one segregating site. Such monomorphism may be a signal of stabilising selection, in particular because our grape samples originated from very diverse geographic regions. Indeed, in grapevine, previous works evidenced a much higher variation rate, with an average of 1 SNP in 47–129 base pairs, according to the genome region and the population studied [52,54,55]. By contrast, the *F* haplotypes for the four sex-linked presented no significant traces of selection, suggesting that these alleles are evolving under a neutral model.

Overall, the sex region presents traits typical of a small XY non-recombining region [21]. According to the commonly accepted model of sex chromosome evolution in plants, such a region can appear in dioecious species when recombination suppression occurs between two closely located male- and female-sterile mutations [22]. The *F* allele is expected to contain a recessive, “loss-of-function” type, male sterility mutation whereas the *M* allele would harbour a fully-functioning male fertility allele with, at a nearby locus, a dominant female sterility mutation [23]. In such a case, the *M* allele is expected to be constrained by selection against a recombination between the two sex-determining loci, since recombination may bring either total sterility, or reversion to the ancestral hermaphrodite state. The accumulation of insertions, inversions, repeated elements and chromosomal rearrangements in the X and the Y counterparts [56] may add to this mechanism, impeding local chromosome pairing at meiosis. Indeed, in this locus, Fechter et al. [18] reported the presence of additional repeated FMO elements and of a retrotransposon in the female allele, both absent from the male allele. These structural differences may help repress local recombination between M and F alleles. The suppression of the recombination may in turn be at the origin of the linkage disequilibrium, and it may as well explain part of the reduction of diversity in *M* alleles.

In this region, the weaker association with the sex trait in a distal (VSVV010) and, if accurately located, a central (VSVV008) genes could be a trace of some recombination events, sufficient to break the association with the sex causal genes, but not ample enough to completely blur LD traces (Figures 2 and 3). Rare recombination events could have prevented the evolution of this small sex region into a full sex chromosome in *Vitis*, although dioecy is supposed to have appeared in this taxon millions of years ago [57]. Finally, if the VSVV008 is well located in the sex locus, sex determinism in *Vitis* might be the result of two distinct sets of mutation in two linked gene regions, one including VSVV006 and VSVV007, and the other including VSVV009. As in *Fragaria virginiana* Mill. [58], the female and male sterile mutations could be not completely linked allowing the appearance of neuter and hermaphrodite individual. Some hermaphrodite grapevines have been already observed in natural conditions, but their wild status is still uncertain today as they may be escapees from cultivation [59]. Similarly, in the long-lived, late-flowering and disease-prone grapes, while non-flowering plants are observed both in the wild and in experimental breeding, it is very difficult to unequivocally establish whether these are neuter or just growing in flowering-limiting conditions.

The length of the small XY region in *Vitis vinifera* is less than 1% of the chromosome length, much shorter than the small sex region identified in papaya which covers 10% of the chromosome [60]. In this small sex region, the *flavin-containing monooxygenase* (FMO) genes and the *adenine phosphoribosyl transferase* (APT3) have been already suggested as good functional candidates for flower sex determination in grapevine [18]. Other candidate genes could be mentioned such as the *trehalose-6-phosphate phosphatase* (TPP) that controls inflorescence architecture in maize through sugar signal modification [61] and its direct product, the disaccharide trehalose, has a marked effect on flowering transition [62]. The *WRKY transcription factors* are one of the largest families of transcriptional regulators [63] and one of these factors has been shown to regulate endosperm growth and cellularization in *Arabidopsis* [64]. The VSVV008 amplicon was designed in a gene which the predicted protein reveals similarity with a *Ethylene Overproducer-like 1* (ETO1). The Arabidopsis ETO1 protein specifically inhibits the enzyme activity of the *1-aminocyclopropane-1-carboxylate synthase* (ACS) [65,66] known to be involved in sex determination in melons (*Cucumis melo*) [67]. However, for the *YABBY* protein, the polymorphisms did not correlate with phenotypic sex, suggesting that the association found by Battilana et al. [20] is the result of an extended intergenic linkage disequilibrium (LD), and not a direct indication of a causal mutation.

### Origin of the H allele and traces of domestication

The last objective of this study was to elucidate the origin of hermaphroditism in domesticated grapevines. Both Fst and network analysis revealed that *H* haplotypes are more closely related to *M* than to *F* haplotypes. Thus, the *H* allele of the domesticated hermaphrodite grapevines may have derived from the *M* allele of wild male grapevines as suggested by previous authors [13,68]. Interestingly, in *Carica papaya,* hermaphrodites are also heterozygous for a Y chromosome variant (Y^h^), more similar to the male-determining Y than to the X [60,69]. However, while all combinations of Y and/or Y^h^ are lethal in Papaya, in *Vitis HH* genotypes do strive and set seeds, as in the case of certain domesticated grapevines such as *Chardonnay*, *Muscat de Hambourg*, *Riesling* or *Cardinal* (Truel pers. comm., Vassal INRA), which produce 100% hermaphrodite progenies. Thus, the *H* allele may be an *M* allele having lost the dominant female sterility mutation, explaining the dominance of the *M* allele over the *H* allele. This hypothesis could also explain the increase in diversity observed in the *H* haplotypes as compared to the M haplotypes.

Studying phylogenetic patterns among the haplotypes, we could only found a weak tendency of the *H* macrohaplotypes of cultivars from Eastern regions cultivars, as compared to Western cvs, to be closer to the wild *M* macrohaplotypes. Former studies situated the major grapevine domestication region in the Eastern part of the Mediterranean area [9,53], which is thus consistent with our data.

More interestingly, the network analysis showed that both the *F* and *M/H* haplogroups are each divided in subgroups. In particular, wild female macrohaplotypes are subdivided in two main groups, one closely connected to the *V. balanseana F* haplotype, and the other farther away from *Vitis* sp. females; domesticated female haplotypes are divided in three groups, the first one close to the *V. balanseana* group, and the other two branching as independent lineages from the main *V. sylvestris* haplogroup. Similarly, in the *M/H* group, while the small differentiation within M haplotypes allows for less discrimination in the wild haplogroup, the cultivated hermaphrodites are again divided in groups, one including Eastern varieties and the other with a Western component. The general picture obtained with the network analysis points to a genetic structure of the wild *V. vinifera* haplotypes, in relation with other species, supporting the hypothesis presented in Peros et al. [25] that two chloroplast lineages from different Asian species (*V. piasezkii*, *V. amurensis* and *V. thunbergii*) contributed to the emergence of wild *V. vinifera* populations in Europe. On the other hand, the group differentiation in the domesticated compartment, both for the *F* and the *H* haplotypes, suggests multiple domestication events, as advanced by Arroyo et al. [11] based on chloroplast genetic diversity. More surprisingly, we found that the *H* haplotype from *cv. Assyl kara*, a Russian cultivar, derives directly, via a series of mutations, from *V. balansaena* (Figure 5). In the *F* group, the cultivar closest to *V. balanseana* is also a Russian cultivar, *cv. Ak ouzioum tapapskii*. Based on this evidence, we can advance the hypothesis that, in the sex region, in addition to the already known contribution from *V. vinifera* ssp. *sylvestris*, domesticated grapes enclose a genetic contribution from different Asian species. It is historically known that during the Soviet Union period, Russian agricultural researchers were active in importing genetic variability from diverse Asian regions as a source of cold or disease resistance alleles [70]. Indeed, Venuti et al. [71] recently showed that the Asian *Vitis amurensis* was used by breeders to introgress resistance genes into cultivated grapevines. However, since in our sample the *cv. Assyl kara* was recorded as one of the oldest traditional cultivar from North Caucasus [72], the introgression of a genetic contribution from Asian species into cultivated grapes may also significantly predate early 20th century breeding activities in Russia. It could well have occurred naturally through gene flow between different interfertile *Vitis* species followed by selection during domestication.

The very small differentiation found between the *H* and *M* haplotypes in the sex-linked amplicons and the small number of individuals studied here makes it difficult to clarify further the domestication pathway; this issue merits without doubt further exploration, reinforcing the argument of Venuti et al. [71] that new prospecting and collection of wild grapes and other *Vitis* species in the Eastern part of the domestication range are strongly needed presently.

The phylogeny position of *V. balansaeana*, *V. coignetiae* and *V. monticola* grapevines in our network, as well as segregation mapping in inter-specific crosses, both support a sex locus shared by all *Vitis* spp. [7,17], suggesting that the development of heteromorphic sex chromosomes is still in the very first stage of evolution in this taxon. In general, the age of a sex-determining region can be estimated from the age of the taxon in which it is found [23]. As in the subgenus *Vitis*, dioecy is the ancestral condition*,* its sex-determining region should be at least as old as the separation of the *Vitis* and *Muscadinia* subgenera, thought to have diverged approx. 18 My ago [57]. Other dioecious species with a sex region of approximately the same age, such as *Silene latifolia* [73], *Bryona dioica* [74] or *Rumex* spp. [75], have reached the final stages of sex chromosome evolution, with either full heteromorphic sex chromosomes or very large regions encompassing hundreds of genes. Future works to fully sequence the sex locus in a larger sample of genotypes in *Vitis* species could contribute to understand why some dioecious plants rapidly developed specific sex chromosomes, while others did not.

## Conclusions

In *Vitis vinifera* subsp. *sylvestris*, we confirmed a sex locus of 151,8 kb located downstream to the marker VVIB23 and displaying haplotype diversity, linkage disequilibrium and differentiation that typically correspond to a small XY sex-determining region with XY males and XX females. This small sex-determining region, spanning less than 1% of chromosome 2 and also present in other *Vitis* species, suggests that grapevines could be organisms of choice to study the early stages of evolution of sex chromosomes in perennial species.

Hermaphrodite alleles appear to derive from male alleles of wild grapevines, with successive recombination events allowing import of diversity from the X into the Y chromosomal region and slowing the expansion of the region into a full heteromorphic chromosome. Macrohaplotypes network patterns are consistent with a major grapevine domestication region in the Eastern part of the Mediterranean area and secondary domestication events in geographically distinct areas. Finally, we hypothesise that in the sex region some domesticated grapes enclose a genetic contribution from different Asian species. Our findings should encourage new prospections and collection of wild grapes, including other *Vitis* species, in the Eastern part of the domestication range.

## Availability of supporting data

The sequences data sets supporting the results of this article are available in the Genbank repository, [GenBank: KJ575622-KJ57662; http://www.ncbi.nlm.nih.gov/genbank/]”.

## Additional files

Additional file 1:
**Passport data of the genotypes studied in the different analyses.** (*) short names used in haplotype trees and the network. Short name signification: c = cultivated; s = wild (sauvage), f = female, m = male, h = hermaprodite, hf or hh = hermaphrodites for which the genotype at the sex locus is predicted from to the study of the sex segregation in their progenies. (**): accessions codes from the INRA Vassal collection, France. (***) Geographic groups acronyms were defined as in Bacilieri et al. [35], namely: MAGH = Maghreb; IBER = Iberian Peninsula; WCEUR = Western & Central Europe; ITAP = Italian Peninsula; BALK = Balkans; RUUK = Russia & Ukraina; EMCA = Eastern Mediteranean and Caucasus; MFEAS = Middle and Far East; NEWO = New World; and ND = Non determined. (#) Geographic origin predicted from molecular evidences and a hierarchical clustering as in Bacilieri et al. [35]. Additional file 2:
**Primer sequences of the amplicons used in this study to cover the sex locus and its edges.** (*) Gaze annotation. (**) Approximative values. (†) PN40024 reference sequence, 12×.0 version, amplicon position 16.072.323-16.073.307, Scaffold_233, chromosome UnRandom. (‡) PN40024 reference sequence, 8× version, amplicon position 5.192.572-5.193.382, scaffold 187, chromosome 2. (§) Primers developed in the gene predicted using the 8x Gaze annotation and confirmed by Fechter et al. [18] on the 12×.0 reference sequence version. Additional file 3:
**Characteristics of the sequence polymorphisms found in the eleven amplicons covering the sex locus.** For the Fisher tests, P-values and -log(p-value) in bold are the polymorphisms significantly deviating from the Hardy-Weinberg equilibrium. The underlined Fisher-test values correspond to significant polymorphisms following perfectly the XY sex-determination model : all male genotypes are heterozygous and all female are homozygous for the most frequent allele, i.e. for example males were A/T and females were A/A but never T/T. (*): the bold and italic position are approximate values; (**) F, M and Th = actual genotype count of females and males in the population, and theoretical proportions at Hardy-Weinberg equilibrium; he = heterozygote genotypes (for example AT). (***): ND = Not Defined. Additional file 4:
**Maximum likelihood haplotypes trees of the four sex-linked gene fragments built to define the** ***M*** **,** ***F*** **and** ***H*** **haplogroups.** Amplicons: a) VSVV006, b) VSVV007, c) VSVV009 and d) VSVV010. According to sex inheritance theory in *Vitis*, the *F* haplotypes regroup the *F* haplotypes of the *FF* females, *MF* males and *HF* hermaphrodites genotypes. Thus, the group containing haplotypes found in female, male and hermaphrodite genotypes was designated as the female *F* haplogoup. In green, haplotypes of domesticated hermaphrodite genotypes, in blue haplotypes of wild male genotypes and in pink haplotypes of wild female genotypes. The red dashed border box indicate the *F* haplogroup. Additional file 5:
**Percentage of heterozygous genotypes in domesticated hermaphrodite grapevine.** a) The gene amplicons’ positions on the sex locus on the grapevine chromosome 2. b) percentage of heterozygous genotypes, at a given polymorphism, in hermaphrodite domesticated grapevines. The 14 genotypes considered here were expected to be heterozygous *HF* at the sex locus considering the sex segregation in their progenies. The red arrows indicate the polymorphisms perfectly linked to sex in male and female wild genotypes (see Figure 2 in the main document). Additional file 6:
**Gene annotation of the sex region defined in the study (Gaze annotation 2012, 12x.0 version).**
Additional file 7:
**Haplotypes of the four sex-linked amplicons.** Haplotype representation for the four sex-linked amplicons ranked according to their inferred sex (from the haplotype trees) and the geographic origin of the genotypes. Haplotypic sex, *M* = male, *H* = hermaphrodite, *F wild* = female haplotype found in wild grapevine and *F dom* = female haplotype found in domesticated grapevine. Name: PHASE haplotype number. Individual: genotype name; short name signification: c = cultivated; s = wild (sauvage), f = female, m = male, h = hermaprodite, hf or hh = hermaphrodite for which the genotype is known (*HF* and *HH* genotype known through the study of the sex segregation in descents). Country: country of origin of the plant. Region: geographic groups acronyms were defined as in Bacilieri et al. [35], namely: MAGH = Maghreb; IBER = Iberian Peninsula; WCEUR = Western & Central Europe; ITAP = Italian Peninsula; BALK = Balkans; RUUK = Russia & Ukraine; EMCA = Eastern Mediterranean and Caucasus; MFEAS = Middle and Far East; NEWO = New World Vineyard; and ND = Not determined. Colors base representation: yellow = adenine, red = thymine, blue = cytosine and green = guanine.
