# Supplementary material for: A small XY chromosomal region explains sex determination in wild dioecious V. vinifera and the reversal to hermaphroditism in domesticated grapevines
Source: BMC Plant Biol. 2014 Sep 3;14:229. doi: 10.1186/s12870-014-0229-z (PMC4167142; doi:10.1186/s12870-014-0229-z)
Supplement: Additional file 4: — Maximum likelihood haplotypes trees of the four sex-linked gene fragments built to define the M , F and H haplogroups. Amplicons: a) VSVV006, b) VSVV007, c) VSVV009 and d) VSVV010. According to sex inheritance theory in Vitis, the F haplotypes regroup the F haplotypes of the FF females, MF males and HF hermaphrodites genotypes. Thus, the group containing haplotypes found in female, male and hermaphrodite genotypes was designated as the female F haplogoup. In green, haplotypes of domesticated hermaphrodite genotypes, in blue haplotypes of wild male genotypes and in pink haplotypes of wild female genotypes. The red dashed border box indicate the F haplogroup. [file 12870_2014_229_MOESM4_ESM.pdf]

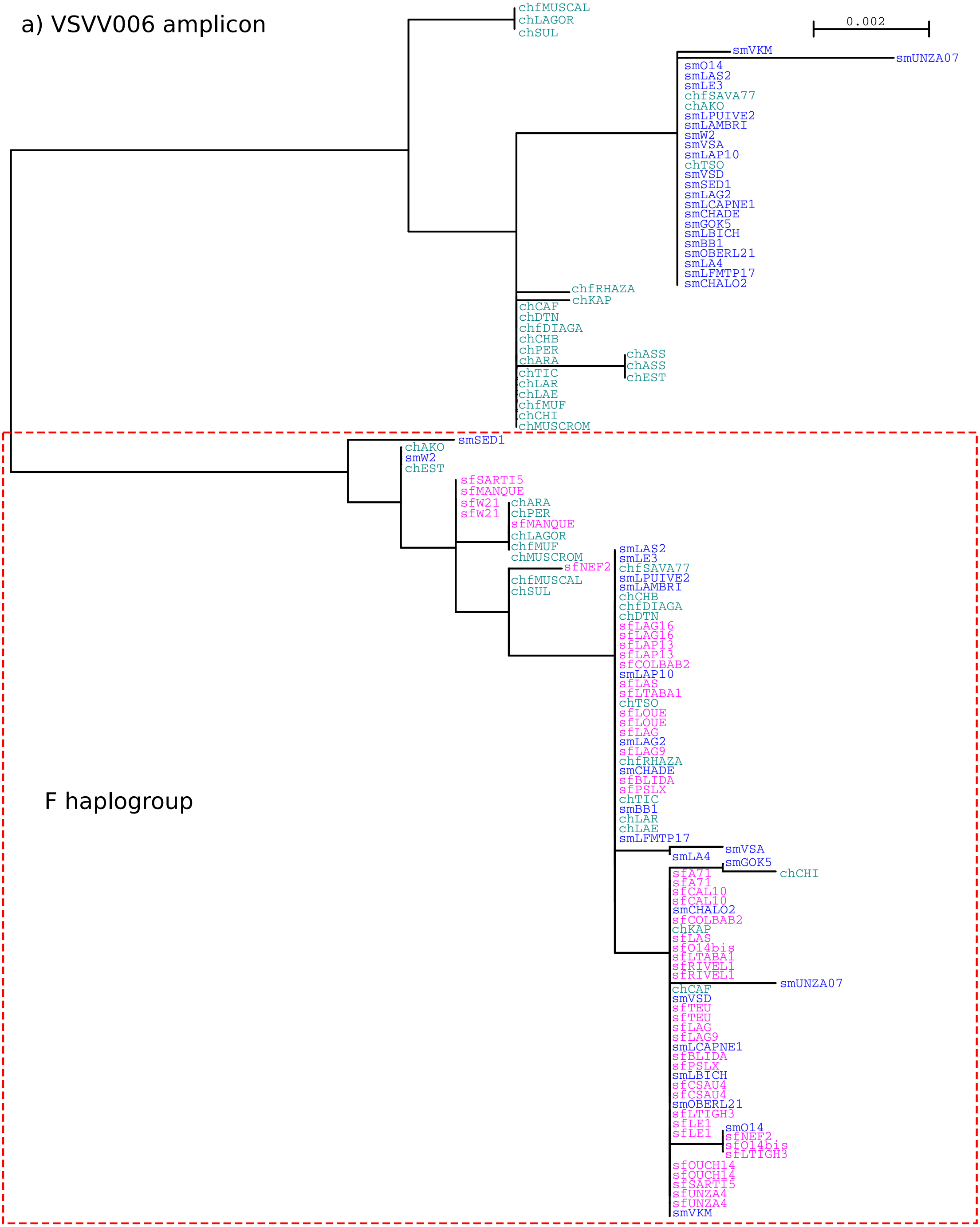

a) VSVV006 amplicon
0.002
chfMUSCAL
chLAGOR
chSUL
smVKM
smUNZA07
smO14
smLAS2
smLE3
chfSAVA77
chAKO
smLPUIVE2
smLAMBRI
smW2
smVSA
smLAP10
chTSO
smVSD
smSED1
smLAG2
smLCAPNE1
smCHADE
smGOK5
smLBICH
smBB1
smOBERL21
smLA4
smLFMTP17
smCHALO2
chfRHAZA
chKAP
chCAF
chDTN
chfDIAGA
chCHB
chPER
chARA
chASS
chASS
chEST
chTIC
chLAR
chLAE
chfMUF
chCHI
chMUSCROM
F haplogroup
smSED1
chAKO
smW2
chEST
sfSARTI5
sfMANQUE
sfW21
sfW21
chARA
chPER
sfMANQUE
chLAGOR
chfMUF
chMUSCROM
sfNEF2
chfMUSCAL
chSUL
smLAS2
smLE3
chfSAVA77
smLPUIVE2
smLAMBRI
chCHB
chfDIAGA
chDTN
sfLAG16
sfLAG16
sfLAP13
sfLAP13
sfCOLBAB2
smLAP10
sfLAS
sfLTABA1
chTSO
sfLOUE
sfLOUE
sfLAG
smLAG2
sfLAG9
chfRHAZA
smCHADE
sfBLIDA
sfPSLX
chTIC
smBB1
chLAR
chLAE
smLFMTP17
smVSA
smLA4
smGOK5
chCHI
sfA71
sfA71
sfCAL10
sfCAL10
smCHALO2
sfCOLBAB2
chKAP
sfLAS
sfO14bis
sfLTABA1
sfRIVEL1
sfRIVEL1
smUNZA07
chCAF
smVSD
sfTEU
sfTEU
sfLAG
sfLAG9
smLCAPNE1
sfBLIDA
sfPSLX
smLBICH
sfCSAU4
sfCSAU4
smOBERL21
sfLTIGH3
sfLE1
sfLE1
smO14
sfNEF2
sfO14bis
sfLTIGH3
sfOUCH14
sfOUCH14
sfSARTI5
sfUNZA4
sfUNZA4
smVKM

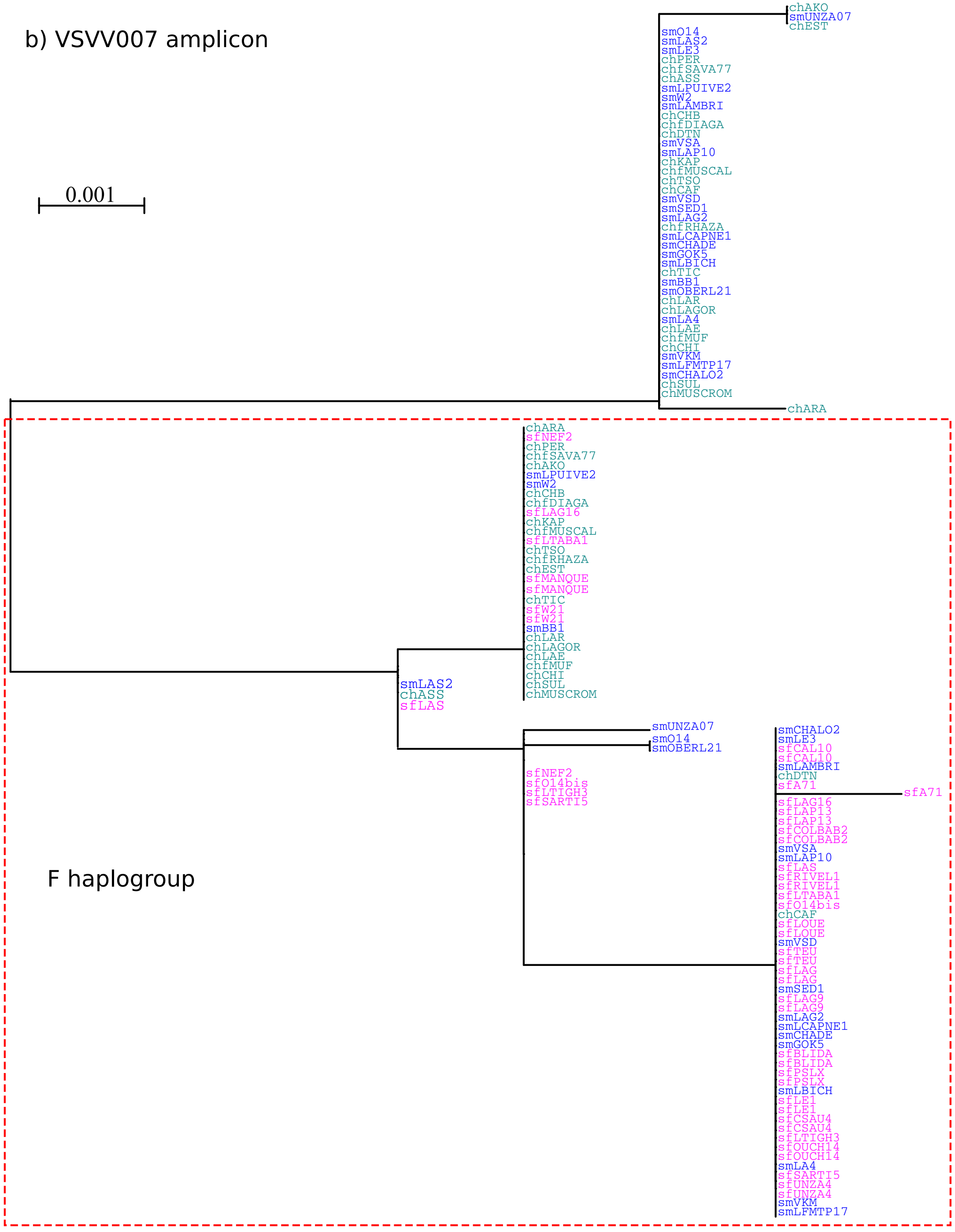
b) VSVV007 amplicon
0.001
chAKO
smUNZA07
chEST
smO14
smLAS2
smLE3
chPER
chfSAVA77
chASS
smLPUIVE2
smW2
smLAMBRI
chCHB
chfDIAGA
chDTN
smVSA
smLAP10
chKAP
chfMUSCAL
chTSO
chCAF
smVSD
smSED1
smLAG2
chfRHAZA
smLCAPNE1
smCHADE
smGOK5
smLBICH
chTIC
smBB1
smOBERL21
chLAR
chLAGOR
smLA4
chLAE
chfMUF
chCHI
smVKM
smLFMTP17
smCHALO2
chSUL
chMUSCROM
chARA
chARA
sfNEF2
chPER
chfSAVA77
chAKO
smLPUIVE2
smW2
chCHB
chfDIAGA
sfLAG16
chKAP
chfMUSCAL
sfLTABA1
chTSO
chfRHAZA
chEST
sfMANQUE
sfMANQUE
chTIC
sfW21
sfW21
smBB1
chLAR
chLAGOR
chLAE
chfMUF
chCHI
chSUL
chMUSCROM
smLAS2
chASS
sfLAS
smUNZA07
smO14
smOBERL21
sfNEF2
sfO14bis
sfLTIGH3
sfSARTI5
smCHALO2
smLE3
sfCAL10
sfCAL10
smLAMBRI
chDTN
sfA71
sfA71
sfLAG16
sfLAP13
sfLAP13
sfCOLBAB2
sfCOLBAB2
smVSA
smLAP10
sfLAS
sfRIVEL1
sfRIVEL1
sfLTABA1
sfO14bis
chCAF
sfLOUE
sfLOUE
smVSD
sfTEU
sfTEU
sfLAG
sfLAG
smSED1
sfLAG9
sfLAG9
smLAG2
smLCAPNE1
smCHADE
smGOK5
sfBLIDA
sfBLIDA
sfPSLX
sfPSLX
smLBICH
sfLE1
sfLE1
sfCSAU4
sfCSAU4
sfLTIGH3
sfOUCH14
sfOUCH14
smLA4
sfSARTI5
sfUNZA4
sfUNZA4
smVKM
smLFMTP17
F haplogroup

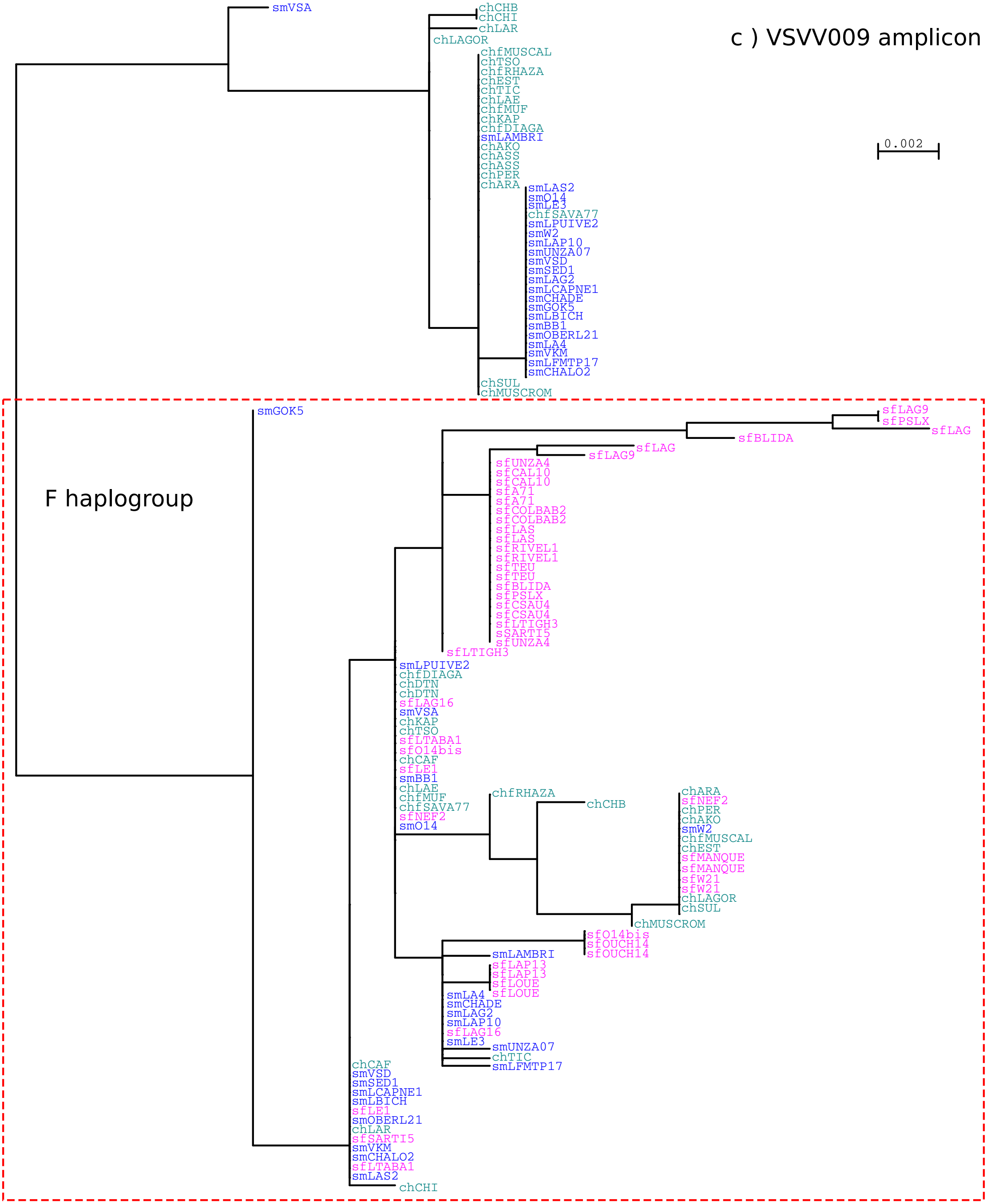

c) VSVV009 amplicon
0.002
smVSA
chCHB
chCHI
chLAR
chLAGOR
chfMUSCAL
chTSO
chfRHAZA
chEST
chTIC
chLAE
chfMUF
chKAP
chfDIAGA
smLAMBRI
chAKO
chASS
chASS
chPER
chARA
smLAS2
smO14
smLE3
chfSAVA77
smLPUIVE2
smW2
smLAP10
smUNZA07
smVSD
smSED1
smLAG2
smLCAPNE1
smCHADE
smGOK5
smLBICH
smBB1
smOBERL21
smLA4
smVKM
smLFMTP17
smCHALO2
chSUL
chMUSCROM
F haplogroup
smGOK5
sfLAG9
sfPSLX
sfLAG
sfBLIDA
sfLAG
sfLAG9
sfUNZA4
sfCAL10
sfCAL10
sfA71
sfA71
sfCOLBAB2
sfCOLBAB2
sfLAS
sfLAS
sfRIVEL1
sfRIVEL1
sfTEU
sfTEU
sfBLIDA
sfPSLX
sfCSAU4
sfCSAU4
sfLTIGH3
sSARTI5
sfUNZA4
sfLTIGH3
smLPUIVE2
chfDIAGA
chDTN
chDTN
sfLAG16
smVSA
chKAP
chTSO
sfLTABA1
sfO14bis
chCAF
sfLE1
smBB1
chLAE
chfMUF
chfSAVA77
sfNEF2
smO14
chfRHAZA
chCHB
chARA
sfNEF2
chPER
chAKO
smW2
chfMUSCAL
chEST
sfMANQUE
sfMANQUE
sfW21
sfW21
chLAGOR
chSUL
chMUSCROM
sfO14bis
sfOUCH14
sfOUCH14
smLAMBRI
sfLAP13
sfLAP13
sfLOUE
sfLOUE
smLA4
smCHADE
smLAG2
smLAP10
sfLAG16
smLE3
smUNZA07
chTIC
smLFMTP17
chCAF
smVSD
smSED1
smLCAPNE1
smLBICH
sfLE1
smOBERL21
chLAR
sfSARTI5
smVKM
smCHALO2
sfLTABA1
smLAS2
chCHI

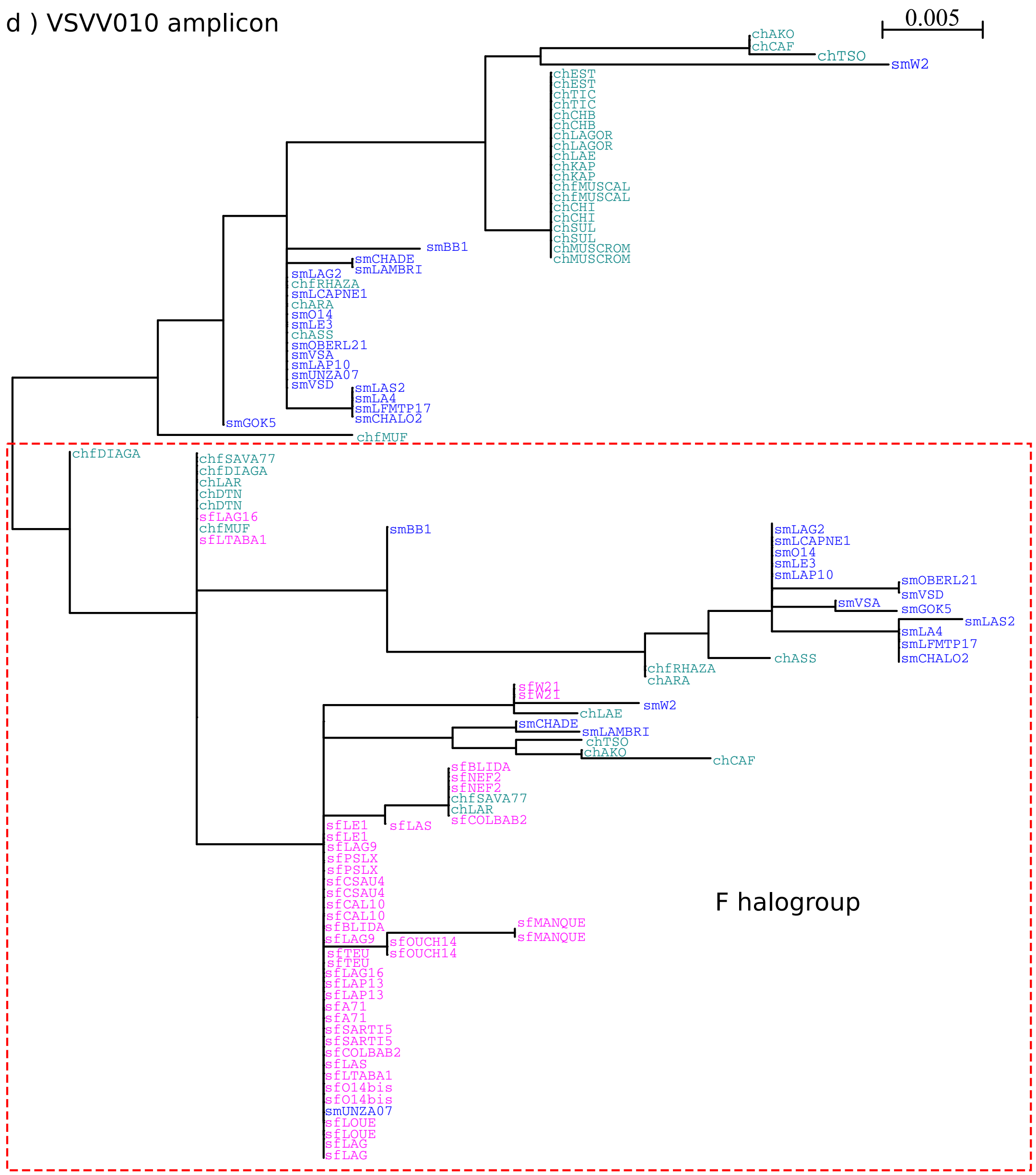
d) VSVV010 amplicon
0.005
chAKO
chCAF
chTSO
smW2
chEST
chEST
chTIC
chTIC
chCHB
chCHB
chLAGOR
chLAGOR
chLAE
chKAP
chKAP
chfMUSCAL
chfMUSCAL
chCHI
chCHI
chSUL
chSUL
chMUSCROM
chMUSCROM
smBB1
smCHADE
smLAMBRI
smLAG2
chfRHAZA
smLCAPNE1
chARA
smO14
smLE3
chASS
smOBERL21
smVSA
smLAP10
smUNZA07
smVSD
smLAS2
smLA4
smLFMTP17
smCHALO2
smGOK5
chfMUF
chfDIAGA
chfSAVA77
chfDIAGA
chLAR
chDTN
chDTN
sfLAG16
chfMUF
sfLTABA1
smBB1
smLAG2
smLCAPNE1
smO14
smLE3
smLAP10
smOBERL21
smVSD
smVSA
smGOK5
smLAS2
smLA4
smLFMTP17
smCHALO2
chASS
chfRHAZA
chARA
sfW21
sfW21
smW2
chLAE
smCHADE
smLAMBRI
chTSO
chAKO
chCAF
sfBLIDA
sfNEF2
sfNEF2
chfSAVA77
chLAR
sfCOLBAB2
sfLAS
sfLE1
sfLE1
sfLAG9
sfPSLX
sfPSLX
sfCSAU4
sfCSAU4
sfCAL10
sfCAL10
sfBLIDA
sfLAG9
sfOUCH14
sfOUCH14
sfMANQUE
sfMANQUE
sfTEU
sfTEU
sfLAG16
sfLAP13
sfLAP13
sfA71
sfA71
sfSARTI5
sfSARTI5
sfCOLBAB2
sfLAS
sfLTABA1
sfO14bis
sfO14bis
smUNZA07
sfLOUE
sfLOUE
sfLAG
sfLAG
F halogroup
