# Supplementary figures and images for: A small XY chromosomal region explains sex determination in wild dioecious V. vinifera and the reversal to hermaphroditism in domesticated grapevines
Source: BMC Plant Biol. 2014 Sep 3;14:229. doi: 10.1186/s12870-014-0229-z (PMC4167142; doi:10.1186/s12870-014-0229-z)

a )

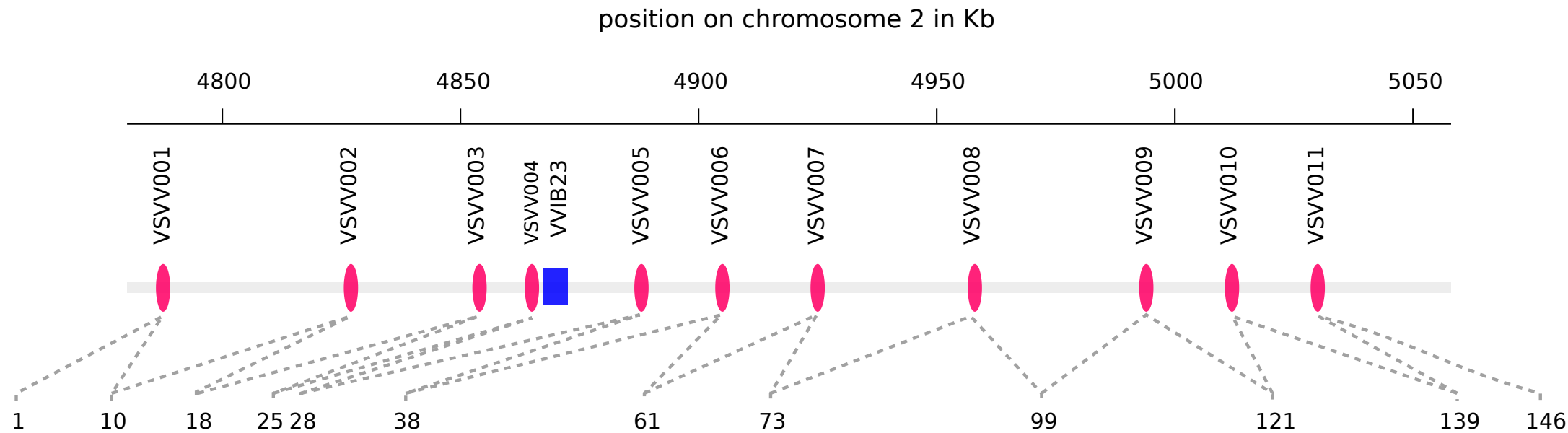

b )

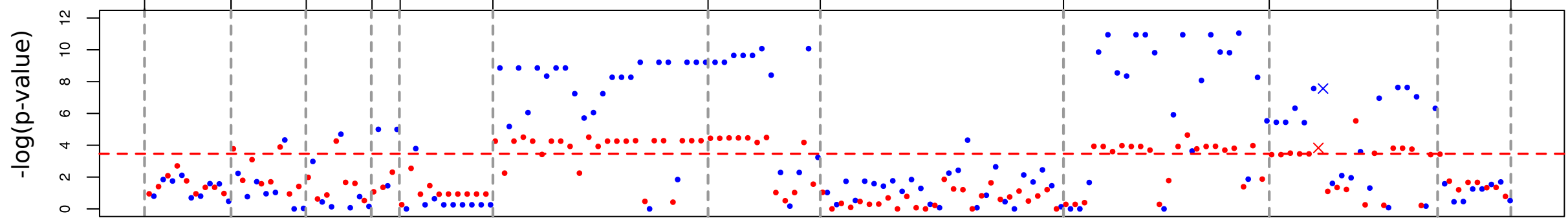

c )

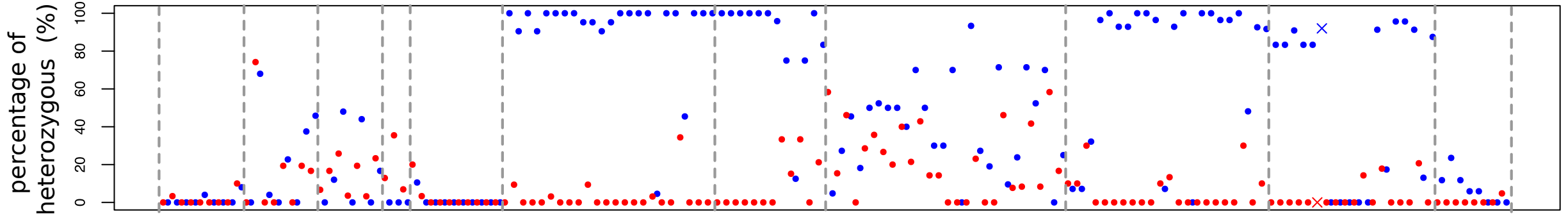

Supplement: Additional file 5: — Percentage of heterozygous genotypes in domesticated hermaphrodite grapevine. a) The gene amplicons’ positions on the sex locus on the grapevine chromosome 2. b) percentage of heterozygous genotypes, at a given polymorphism, in hermaphrodite domesticated grapevines. The 14 genotypes considered here were expected to be heterozygous HF at the sex locus considering the sex segregation in their progenies. The red arrows indicate the polymorphisms perfectly linked to sex in male and female wild genotypes (see Figure 2 in the main document). [file 12870_2014_229_MOESM5_ESM.pdf]
